# Comparison of Proliferation and Genomic Instability Responses to *WRN* Silencing in Hematopoietic HL60 and TK6 Cells

**DOI:** 10.1371/journal.pone.0014546

**Published:** 2011-01-18

**Authors:** Xuefeng Ren, Sophia Lim, Zhiying Ji, Jessica Yuh, Vivian Peng, Martyn T. Smith, Luoping Zhang

**Affiliations:** Genes and Environment Laboratory, Division of Environmental Health Sciences, School of Public Health, University of California, Berkeley, California, United States of America; The University of Hong Kong, Hong Kong

## Abstract

**Background:**

Werner syndrome (WS) results from defects in the RecQ helicase (WRN) and is characterized by premature aging and accelerated tumorigenesis. Contradictorily, WRN deficient human fibroblasts derived from WS patients show a characteristically slower cell proliferation rate, as do primary fibroblasts and human cancer cell lines with WRN depletion. Previous studies reported that WRN silencing in combination with deficiency in other genes led to significantly accelerated cellular proliferation and tumorigenesis. The aim of the present study was to examine the effects of silencing *WRN* in p53 deficient HL60 and p53 wild-type TK6 hematopoietic cells, in order to further the understanding of WRN-associated tumorigenesis.

**Methodology/Principal Findings:**

We found that silencing *WRN* accelerated the proliferation of HL60 cells and decreased the cell growth rate of TK6 cells. Loss of WRN increased DNA damage in both cell types as measured by COMET assay, but elicited different responses in each cell line. In HL60 cells, but not in TK6 cells, the loss of WRN led to significant increases in levels of phosphorylated RB and numbers of cells progressing from G1 phase to S phase as shown by cell cycle analysis. Moreover, WRN depletion in HL60 cells led to the hyper-activation of homologous recombination repair via up-regulation of RAD51 and BLM protein levels. This resulted in DNA damage disrepair, apparent by the increased frequencies of both spontaneous and chemically induced structural chromosomal aberrations and sister chromatid exchanges.

**Conclusions/Significance:**

Together, our data suggest that the effects of *WRN* silencing on cell proliferation and genomic instability are modulated probably by other genetic factors, including p53, which might play a role in the carcinogenesis induced by WRN deficiency.

## Introduction

Werner syndrome helicase (WRN), the protein defective in Werner syndrome (WS) patients [1], belongs to the RecQ family of helicases, which are conserved from *Escherichia coli* to humans [2]. WRN has been shown to interact physically and functionally with a number of cellular proteins, subsequently involving it in many aspects of DNA metabolic processes including DNA repair, recombination, transcription and replication [3], [4], [5], [6]. WS carries an enhanced risk of neoplasmas of mesenchymal origin [7], [8]. Recent work has indicated that the role of WRN in human pathogenesis may be much broader than envisaged before, and goes beyond the WS. The polymorphisms of *WRN* gene is associated with increased risks of cancer development, including but not limit to breast, gastric adenocarcinoma and bone and soft tissue sarcomas [9], [10], [11], [12]. In addition, *WRN* gene is inactivated by methylation in a large fraction of common sporadic epithelial malignancies [13]. Understanding how the WRN deficiency leads to a rapid heritable and sporadic carcinogenesis thus becomes a critical task relevant to the new forms of treatment and prognosis of cancer.

WRN has been suggested to be the “caretaker” of the genome [14], as its absence in WS patients leads to increased genomic instability and predisposition to cancer. Interestingly however, studies have continuously found that WRN deficient human fibroblasts derived from WS patients show a characteristically slower cell proliferation rate [15], [16]. Acute depletion of WRN in primary fibroblasts [17] and human cancer cell lines [18] led to marked growth inhibition. These contradictory observations that WRN loss in WS patients leads to increased and accelerated tumorigenesis while cells with WRN deficiency inhibit tumor cell growth suggests a complex role for WRN in tumorigenesis.

We recently reported that cellular proliferation is significantly accelerated after silencing *WRN* in *p53* null hematopoietic HL60 cells [19], providing support for the idea that the loss of WRN could lead to rapid growth and hence tumorigenesis in certain situations. This was supported by the findings from *wrn*-knockout mice studies in which knockout of *wrn* alone did not lead to WS phenotype while crossing *wrn*-knockout mice with *p53*-null mice or mice carrying a null mutation in the *terc* gene, resulted in classic WS phenotypes: premature death, rapid tumorigenesis, etc. [20], [21], [22], [23].

The aims of the present study were to further examine the biological effects of silencing *WRN* in HL60 cells, as well as in *p53* wild-type hematopoietic TK6 cells. As reported here, silencing *WRN* in HL60 cells stimulates RB phosphorylation, driving cells from G1 to S phase and accelerating proliferation. We further demonstrate that *WRN* depletion leads to hyper-activated homologous recombination repair via up-regulation of RAD51 and BLM, which in turn results in DNA damage disrepair and chromosomal aberrations. These effects are not observed in TK6 cells deficient in WRN, indicating that differences in the status of p53 or other factors between these two cell types might play a role in the carcinogenesis induced by WRN deficiency. This suggests that the effects of WRN deficiency on cell proliferation and genomic instability are modulated by other factors.

## Results

### The depletion of *WRN* by shRNA has different effects on the cell proliferation rates of HL60 and TK6 cells

**Figure 1:**
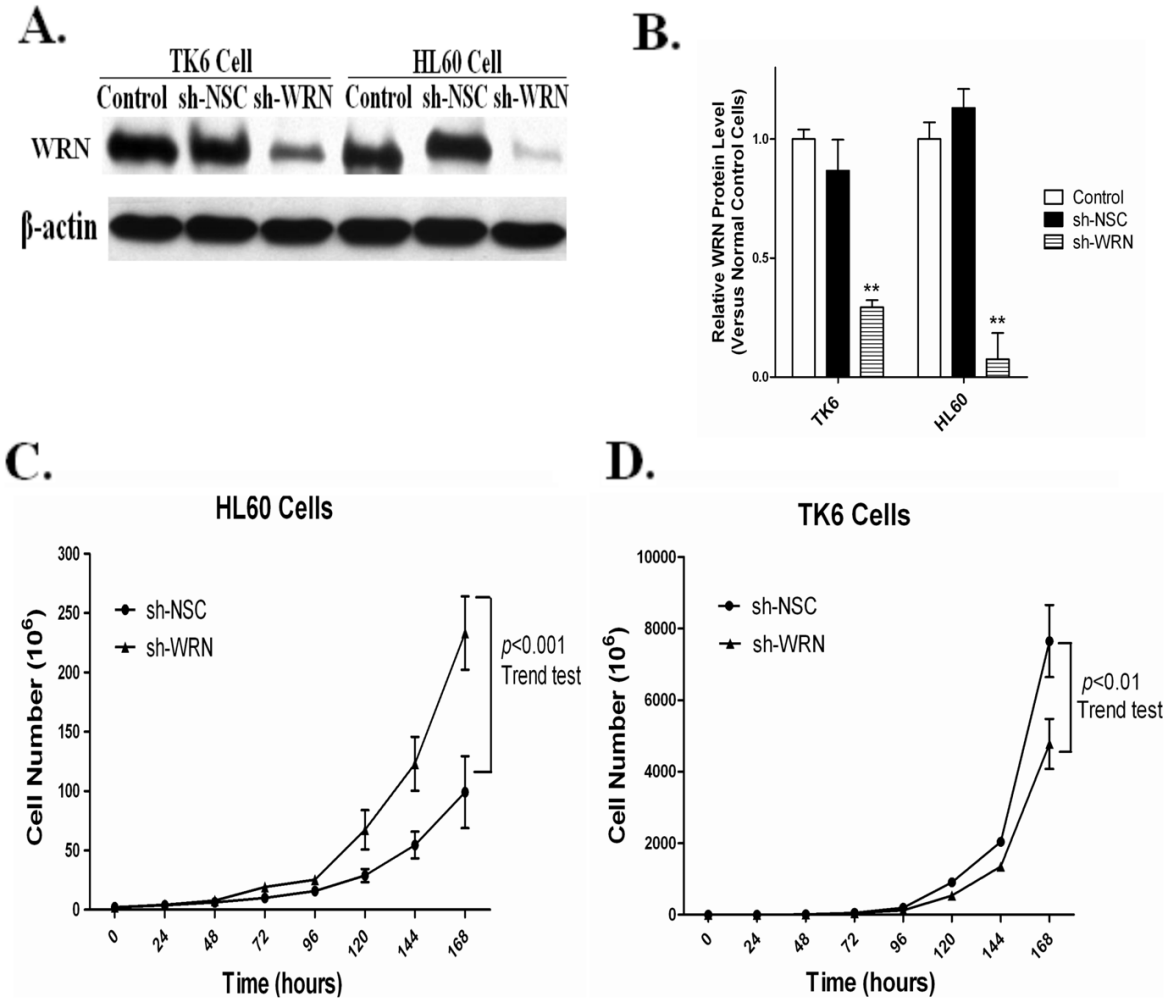
Knockdown of *WRN* expression in HL60 and TK6 cells leads to altered cell proliferation rates. (A) Immunoblot analysis shows the specific decrease of WRN protein in HL60 and TK6 cells transduced with shRNA constructs targeting *WRN*. (B) WRN levels were measured and normalized to β-actin. The values presented are mean + SE (n = 3) and ** indicates *p* value <0.01. (C & D) WRN deficient HL60 and TK6 cells and their relative control cells were cultured over a seven-day period and cell proliferation rates were evaluated using a hemocytometer and the trypan blue exclusion assay. Total cell numbers were plotted against day of initiation. In contrast to HL60 sh-WRN cells that displayed a significantly accelerated proliferation rate (Panel C, Adapted from Ren et al., 2009), the proliferation rate of TK6 sh-WRN cells was slightly decreased (Panel D). The data represents the average of three independent experiments, in which a trend test was performed (*p*<0.01).

To investigate the effects of silencing WRN in human hematopoietic cells, we used lentiviral-based vectors to knock down the protein WRN in both HL60 and TK6 cells (Figure. 1A) with modification from Ren et al, 2009 [19]. The expression of WRN protein was significantly reduced by more than 90% in the HL60 sh-WRN cells and by approximately 75% in the TK6 sh-WRN cells (Figure. 1B). HL60 sh-WRN cells displayed a significantly accelerated proliferation rate over seven days of culture compared to control HL60 sh-NSC cells as evidenced by the greater than 2.5 fold increase of total cell number by the end of the culturing period (Figure. 1C) [19]. In contrast, the proliferation rate of TK6 sh-WRN cells was slightly decreased such that the resultant total cell numbers were about 30% less than the control TK6 sh-NSC cells (Figure. 1D). The growth characteristics of the control cells remained unaltered by the non-silencing shRNA in both cell types (Data not shown).

### Accelerated proliferation rate in HL60 cells is associated with an increased fraction of cells in S-phase partially due to RB-hyperphosphorylation

**Figure 2:**
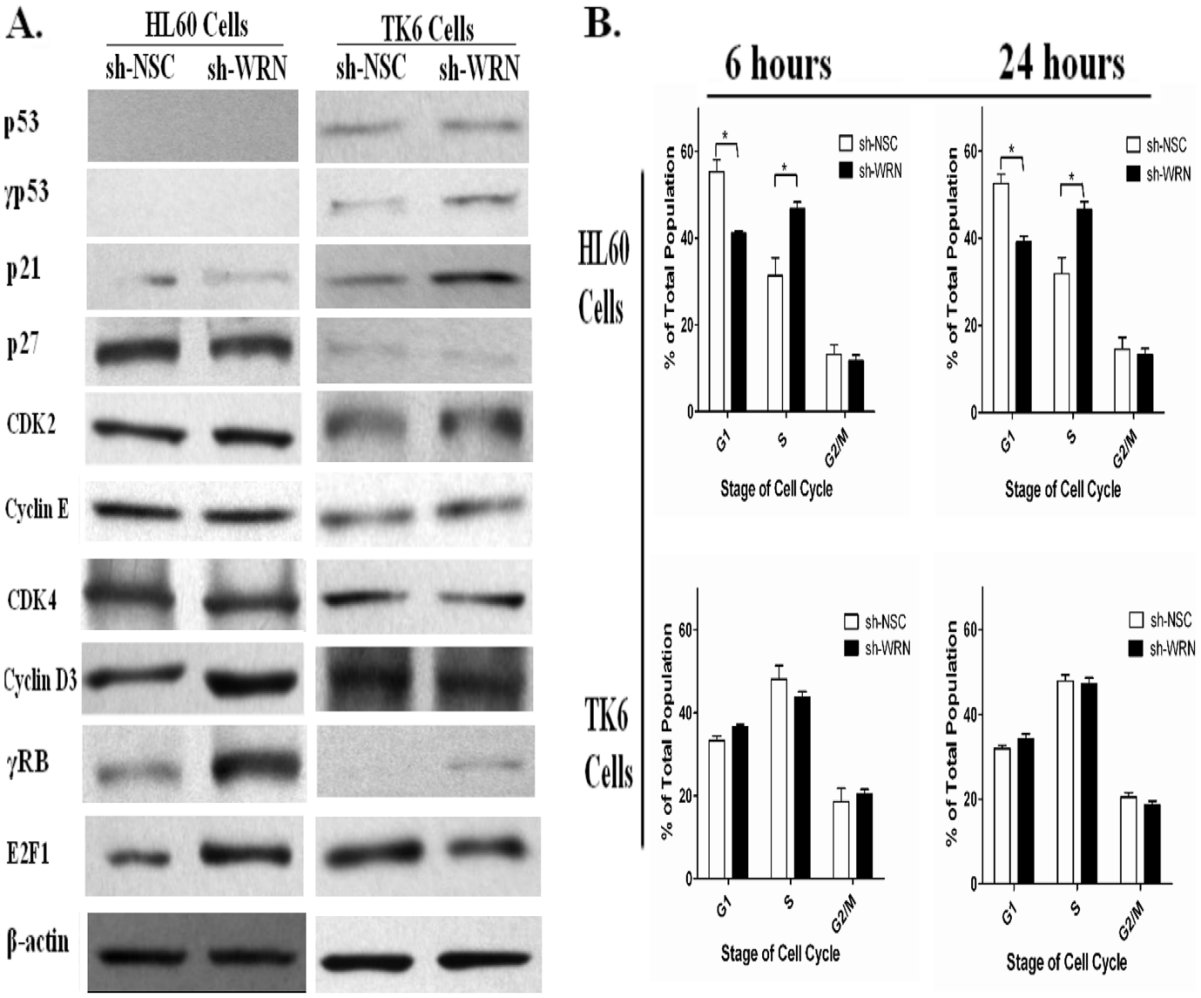
Silencing *WRN* increases γRB protein level and changes the cell replication rate in HL60 cells. (A) Total cell lysates from HL60 sh-WRN, HL60 sh-NSC, TK6 sh-WRN and TK6 sh-NSC cells were subjected to immunoblot analysis with indicated antibodies. Western blots are representative of three independent experiments. (B) Quantification analysis of the cell cycle distribution was performed by flow cytometric based methods. WRN loss increased the fraction of HL60 cells in S phase from 32% in HL60 sh-NSC cells to approximately 43% in HL60 sh-WRN cells across all time points. In contrast, the cell cycle distribution remained comparable between WRN deficient TK6 cells and control cells.

Given the altered proliferation pattern between HL60 and TK6 cells, we measured levels of key proteins in p53 regulated G1/S phase cell cycle checkpoint pathway. As expected, neither total nor phosphorylated p53 protein, were detected in the control and WRN deficient HL60 cells, and p21 protein levels remained consistently low (Figure. 2A). However, levels of phosphorylated RB (γRB) were significantly increased in WRN deficient HL60 cells compared to the control HL60 cells. RB serves as the gatekeeper for progression through the proliferation restriction point (R point) of the cell cycle [24], blocking progression in its hypophosphorylated state. Hyperphosphorylation of RB, together with other factors, allows G1-S progression, and increases the rate of cell proliferation. Examination of the levels of cyclin D/CDK4 and cyclin E/CDK2, thought to be responsible for the phosphorylation of RB, revealed higher levels only of cyclin D3 in HL60 sh-WRN cells compared to control cells, with CDK2, CDK4 and cyclin E remaining unaltered (Figure. 2A). In contrast, the levels of phosphorylated p53, as well as p21 were slightly increased, and subsequently resulted in decreased E2F1 level in TK6 sh-WRN cells compared to TK6 sh-NSC cells (Figure. 2A).

We determined whether the variation in cell cycle related proteins induced by WRN deficiency in the two cell lines, reflected differences in the cell cycle distribution between WRN deficient cells and their control cells. Under standard growth conditions, the S phase fraction was much higher in control TK6 cells when compared to control HL60 cells, approximately 45% versus 30% respectively (Figure. 2B). This is consistent with the fact that the doubling time for the TK6 cells is only 14 hours compared to 27 hours for the HL60 cells (Data not shown). The loss of WRN led to a significant increase in the fraction of HL60 cells in S phase to 43%, from 32% in HL60 sh-NSC cells, across all time points (Figure. 2B), indicating an association between the accelerated proliferation rate in HL60 sh-WRN cells and accelerated DNA synthesis. In contrast, knocking down WRN in TK6 cells had very marginal effects, and the cell cycle distribution remained comparable between WRN deficient TK6 cells and control cells (Figure. 2B).

### HQ exposure enhances DNA damage in *WRN* deficient HL60 cells but induces comparable cytotoxicity between control and *WRN* deficient HL60 cells

Several lines of evidence support the view that WRN plays important roles in both unperturbed DNA replication [25], [26] and the response to DNA damage, more specifically the repair of DNA double-stand breaks (DSBs) via HR [27], [28]. We thus treated both HL60 and TK6 cells with hydroquinone (HQ), a confirmed DSB inducer [29], as a means to further compare the effects of WRN deficiency on the response to DNA damage in HL60 and TK6 cells.

**Figure 3:**
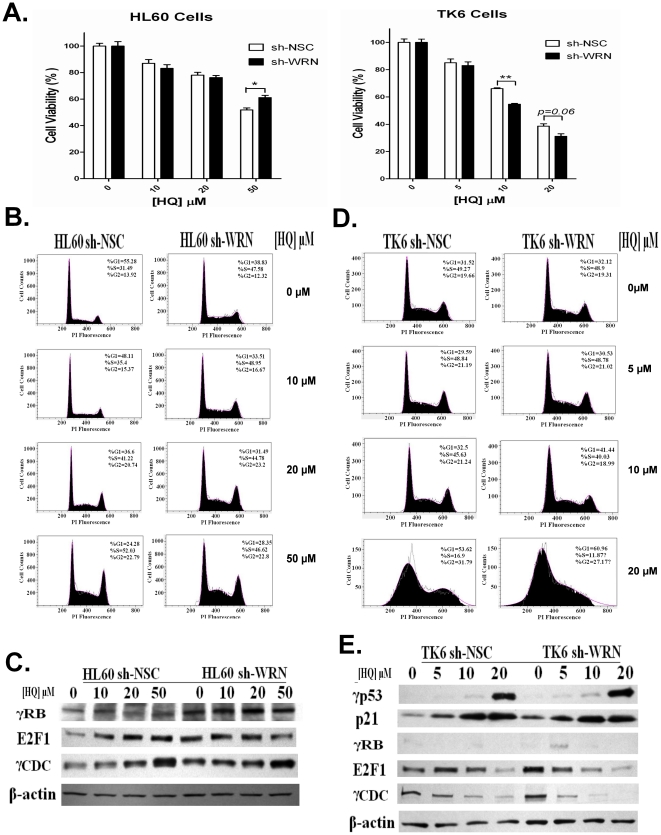
Silencing *WRN* leads to a differential response to HQ treatment between HL60 and TK6 cells. (A) Cells were exposed to HQ for 24 hours and the cell number and viability were calculated using a hemocytometer and the trypan blue exclusion assay. (B) Cell cycle analysis showed HQ exposure induced a dose-dependent decrease of G1-phase and an increase of cells in S and G2 phase in HL60 sh-NSC cells. In contrast, HQ-induced DNA damage in HL60 sh-WRN cells led to a dose-dependent increase of G2 phase, while the proportion of cells in S phase was unchanged despite HQ exposure. (C) HQ treatment induces a dose-dependent increase of phosphorylated CDC2, indicating the failure of cells to protect genomic integrity during DNA replication. Total cell lysates from HL60 sh-NSC and HL60 sh-WRN cells were subjected to immunoblot analysis with indicated antibodies. Western blots are representative of three independent experiments. (D) HQ treatment led to a dose-dependent increase in the fraction of cells in G1 phase and decrease in S phase in both TK6 sh-NSC and TK6 sh-WRN cells. (E) HQ-induced DNA damage activated p53 regulated G1/S phase cell cycle checkpoint pathway in TK6 cells. Immunoblot analysis showed that the increase of the protein levels of γp53 and p21 and the decrease of E2F1 and γCDC2 protein levels were observed with HQ exposure, which were not affected by the loss of WRN.

Using previously identified non-toxic and mildly toxic concentrations of HQ, treatments induced dose-dependent cytotoxicity in both cell types regardless of WRN status (Figure. 3A). Surprisingly however, the loss of WRN in HL60 cells resulted in a slight but significant resistance to HQ toxicity at the highest treatment group (i.e 20 µM) while the loss of WRN in TK6 cells led to increased cytotoxicity after HQ treatment at this dose (Figure. 3A). We then assessed the effects of WRN depletion on DNA damage with or without HQ treatment using the alkaline COMET assay. Loss of WRN in HL60 cells induced a significant increase in DNA damage as measured by % tail DNA, tail intensity and tail moment in the COMET analysis (Table 1). In contrast, silencing *WRN* in TK6 cells had a relatively small effect, as TK6 sh-WRN cells exhibited only a slightly higher level of endogenous DNA damage than TK6 sh-NSC cells (Table 2). Furthermore, HQ treatment in both cell types led to significant and dose-dependent DNA damage regardless of WRN status (Table 1 & 2). However, as shown in table 1, all three measured parameters were significantly increased in the WRN deficient HL60 cells for all doses. In contrast, the effects of the loss of WRN in TK6 cells were only seen in the middle dose group (Table 2).

**Table 1:** DNA damage in HL60 cells treated with HQ for 6 hours analyzed by COMET assay.

			HL60 sh-NSC			HL60 sh-WRN	
Mean		Tail	Tail	% Tail	Tail	Tail	% Tail
(± STM)		Length	Moment	DNA	Length	Moment	DNA
[HQ]µM	0	1.22±0.26	0.14±0.04	3.29±0.24	1.94**±0.22	0.33**±0.04	6.10*±0.49
	10	2.31±0.67	0.50±0.32	3.49±0.39	6.64**±1.08	1.35**±0.37	9.22**±0.74
	20	7.98±0.94	2.43±0.46	9.81±0.60	17.30**±1.14	4.92**±0.42	19.37**±0.80
	50	10.46±0.83	2.74±0.29	15.31±0.72	24.52**±1.26	9.77**±0.64	28.43**±0.86

Note: Mann-Whitney test was used to compare DNA damage between HL60 sh-WRN cells and HL60 sh-NSC cells (*p value <0.05, **p value <0.01).

**Table 2:** DNA damage in TK6 cells treated with HQ for 6 hours analyzed by COMET assay.

			TK6 sh-NSC			TK6 sh-WRN	
Mean		Tail	Tail	% Tail	Tail	Tail	% Tail
(± STM)		Length	Moment	DNA	Length	Moment	DNA
[HQ]µM	0	10.14±1.08	3.75**±0.43	11.50±1.17	9.53±0.84	2.48±0.31	13.54*±0.75
	5	14.41±1.23	6.83±0.81	17.20±1.15	19.66**±1.34	8.37*±0.84	23.68**±1.09
	10	24.04±1.51	10.58±0.94	22.84±1.24	46.08**±1.86	22.42**±1.29	33.83**±1.24
	20	69.23±2.48	46.98±2.47	58.34±1.78	66.77±1.44	44.47±1.36	61.07±0.99

Note: Mann-Whitney test was used to compare DNA damage between TK6 sh-WRN cells and TK6 sh-NSC cells (*p value <0.05, **p value <0.01).

Further analysis showed that HQ-induced DNA damage led to a dose-dependent decrease in the number of cells in G1-phase and an increased number of cells in S and G2 phase in HL60 sh-NSC cells (Figure. 3B). By contrast, HQ treatment further reduced the cells in G1 phase and resulted in a dose-dependent increase of G2 phase in HL60 sh-WRN cells, but did not alter the proportion of cells in S phase (Figure. 3B). As the primary role of activated p53 is to inhibit the progression from G1 to S phase in the presence of DNA damage [30], it was suspected that the deficiency of p53 in HL60 cells would result in the loss of this checkpoint function in the G1-S phase, which was supported by our results (Figure. 3B) and reinforced by the changes in the cell cycle checkpoint protein levels (Figure. 3C). The decreased fraction of cells in G1 phase was associated with increased levels of γRB and E2F1 (Figure. 3C), thus signaling the ability to undergo replication despite the presence of DNA-damage in HL60 cells. As WRN is required for correct recovery of DNA damage induced during S-phase of the cell cycle [31], an increased G2 phase companied with an hyperphosphorylated CDC2 (Figure. 3C) indicates the failure of cells to protect genomic integrity during DNA replication [32].

In TK6 cells, DNA damage induced by HQ treatment led to a dose-dependent increase in the fraction of cells in G1 phase and a decreased fraction in S phase in both TK6 sh-NSC and TK6 sh-WRN cells, as the suppression of WRN did not alter the distribution of cells in each cell cycle phase (Figure. 3D). The cell cycle pattern results seen in TK6 cells were well reinforced by the activated p53 regulated G1/S phase cell cycle checkpoint pathway, which apparently was not affected by the loss of WRN (Figure. 3E).

### Loss of *WRN* activates the Rad51-HR repair pathway and leads to DNA damage disrepair and elevated chromosomal aberrations in HL60 cells

**Figure 4:**
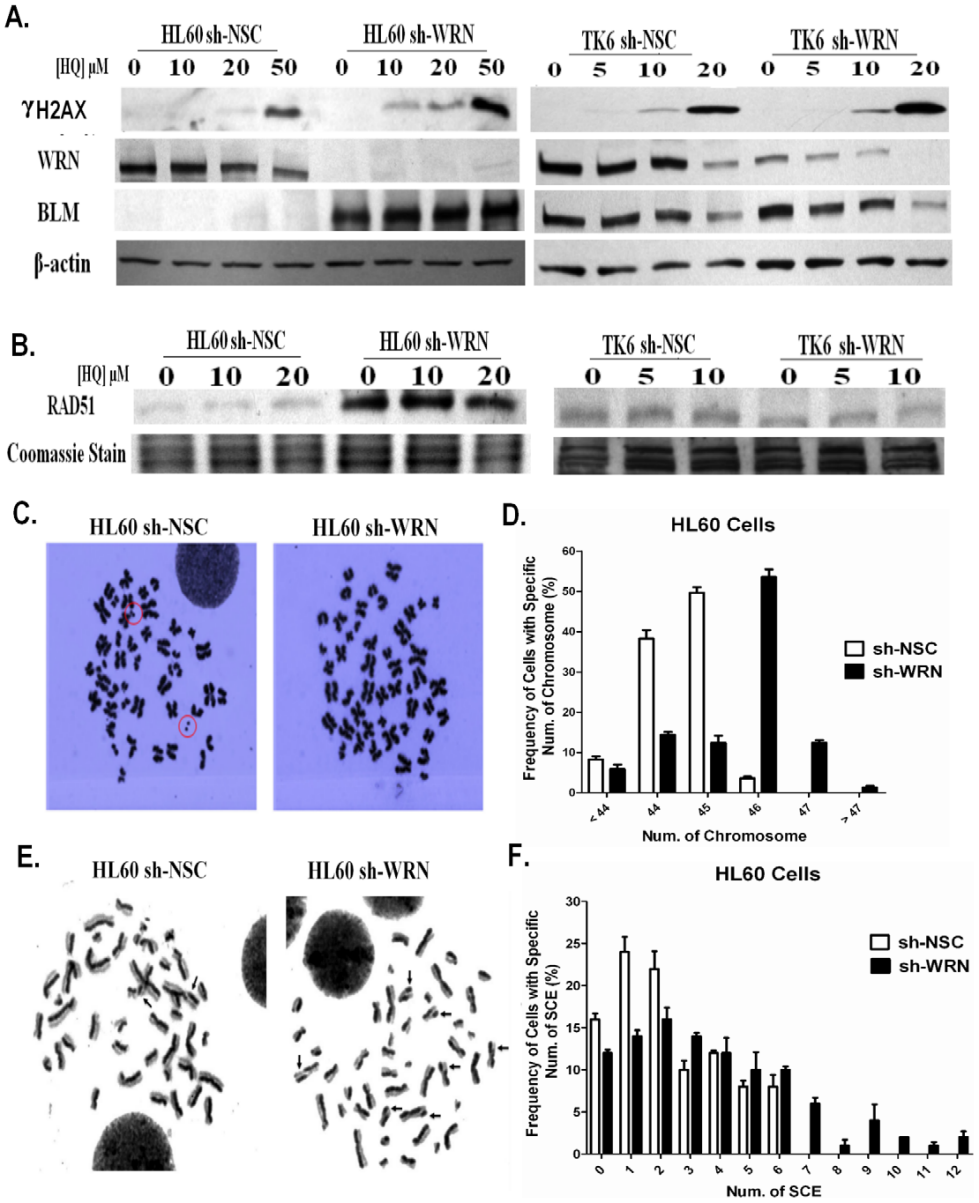
WRN depletion in HL60 cells leads to hyper-activated HR, DNA damage disrepair and chromosomal aberrations. (A & B) Representative images of immunoblot analysis performed using antibodies against γH2AX, WRN, BLM in whole cell lysate and RAD51 on nuclear extracts. Immunoblot analysis showed that accumulation of γH2AX in cells exposed to HQ for 24h in a dose-dependent manner. BLM and RAD51 dramatically increased in HL60 sh-WRN cells compared to HL60 sh-NSC cells regardless of HQ treatment. WRN depletion in TK6 cells slightly reduced RAD51 protein levels. (C) Double minutes (marked with red circle), the characteristic chromosomal aberration in HL60 cells, were absent in HL60 sh-WRN cells regardless of HQ treatment. (D) Aneuploidy in WRN deficient HL60 cells resolved to 46 chromosomes versus 44 or 45 typical of control HL60 cells. (E) Representative images of SCE in HL60 sh-NSC and HL60 sh-WRN cells are shown (marked with arrow). (F) Cells with a higher frequency of SCE are observed in HL60 sh-WRN when compared to the HL60 sh-NSC cells.

We measured phosphorylated H2AX levels, a marker of DNA DSBs and an essential component of DSB repair machinery [33], in both WRN knockdown and control cells, after HQ treatment. As shown in Figure 4A, a dose-dependent increase in γH2AX levels was observed in all measured cells 24 hours after HQ exposure. However, γH2AX levels were higher in the WRN deficient cells, particularly the HL60 cells (Figure. 4A), in which γH2AX was detected even at the lowest dose of HQ.

The HR signaling pathway, in which WRN is involved, is highly regulated and dependent on RAD51 [34] and its interactions with a number of other co-factors such as WRN, BLM, p53, *etc.* to maintain genomic stability [35]. We showed that loss of WRN in HL60 cells leads to a dramatic increase in RAD51 and BLM protein levels regardless of HQ treatment (Figure. 4 A&B), suggesting that HR is constitutively elevated in response to the increased DSBs in WRN deficient HL60 cells and may be occurring through compensation mediated by another helicase, BLM. However, the protein levels of nuclear RAD51 and BLM were slightly reduced in the TK6 cells, and HQ treatment resulted in no changes on either RAD51 or BLM levels (Figure. 4 A&B).

WS is a classic chromosomal instability syndrome, and cells isolated from WS patients demonstrate increased chromosomal aberrations [36], [37], [38]. A higher frequency of HR has been implicated in inducing chromosomal aberrations, and thus carcinogenesis [39], [40]. As demonstrated in Figure 4C, elevated levels of HR in WRN deficient HL60 cells resulted in increased levels of chromosomal damage repair, completely changing the cytogenetic characteristics of HL60 cells, and resulting in both the disappearance of the characteristic double minutes and the reduction in the total number of cells with abnormal chromosomal numbers (Figure. 4 C&D and Figure. S1). As such, aneuploidy was not as prevalent in WRN deficient HL60 cells as compared to control cells (Figure. 4D). Interestingly, HQ treatment also led to effects similar to WRN deficiency, such as a dose-dependent reduction of double minute chromosomes and aneuploid cells (Figure. S1 and Table S1). Both effects may be related to the reduction of WRN levels as indicated by the reduction of WRN levels with increasing HQ doses in control cells (Figure. 4A). However, we also observed that loss of WRN in HL60 cells resulted in increased amounts of structural chromosomal aberrations when compared to control HL60 sh-NSC cells (Table S1). Similarly, HL60 sh-WRN cells displayed a higher frequency of sister chromatid exchange (SCE) than the HL60 sh-NSC cells (Figure. 4 E&F). The frequency of SCE averaged to be 3.6 SCE per cell in HL60 sh-WRN cells versus 2.3 SCE per cell in the HL60 sh-NSC cell (Data not shown). Sister chromatid exchange (SCE) has been linked to disrepair resulting from the imbalance of HR activity [41], [42]. In contrast, WRN deficiency alone had little effect in TK6 cells in terms of the non-banding chromosomal analysis. Rather, corresponding with the impaired HR activity resulting from HQ treatment in TK6 cells, the amount of abnormal chromosomal structures increased with HQ treatment (Table S1), implying decreased activation of repair mechanisms.

## Discussion

WRN deficiency in WS patients leads to rare premature aging syndromes associated with an excess of unusual cancer types [7], [43] including soft tissue sarcomas, thyroid cancers, and meningiomas [44]. As the mechanism of how *WRN* mutations lead to cancer in WS patients is still not entirely understood, researchers continue to pursue a better understanding of how these cancers evolve. We were especially interested in understanding how WRN deficient cells are able to overcome their characteristic slower proliferation rate [15], [16] and induce tumorigenicity [7], [44].

We previously reported that acute silencing of *WRN* significantly accelerated the growth rate of HL60 cells [19]. Although an *in vivo* study reported that the loss of WRN in p53 null background mice led to rapid tumorigenesis [20], this is the first time to our knowledge that the silencing of *WRN* has been shown to promote cell growth *in vitro*. Here, we show that the loss of WRN leads to a dramatic increase in the growth rate of p53-deficient HL60 cells (Figure. 1), while its loss in TK6 cells leads to a decreased proliferation rate consistent with other WRN deficient cell lines [15], [16]. Under normal culturing conditions, TK6 cells grow faster than HL60 cells, consistent with the observation that the majority of control TK6 cells were in the replication phase (S phase), while the majority of the HL60 cells were in G1 phase (Figure. 2). Loss of WRN in HL60 cells resulted in a significantly increased fraction of cells in S phase, suggesting that the rapid accumulation of cells in replication phase and the distinct cell cycle distribution pattern contributed to the increased proliferation rate in WRN deficient HL60 cells.

Eukaryotic cells have evolved a collection of complex networks for the regulation of the cell cycle, in which cell cycle checkpoints are crucial to preventing cells with damaged DNA from proceeding to the next phase, allowing verification of necessary phase processes, and repairing DNA damage [45]. Dysregulation of the cell cycle components may cause the cell to uncontrollably multiply or lead to tumorigenesis. While there are several checkpoints within the cell cycle, the G1/S transition is the rate-limiting step that plays a central role in deciding whether the cell should divide, delay division, or enter a quiescent stage, which in turn determines the cell proliferation rate. P53 and RB are essential for the G1/S cell cycle checkpoint [46], [47]. As expected, the p53 mediated DNA repair pathway was activated in response to increased spontaneous DNA damage in WRN deficient TK6 cells, which resulted in delayed progression of cells into S phase such that slower population growth followed. In contrast, in HL60 cells, the data not only confirmed that p53 regulated G1/S checkpoint proteins were deficient, but also demonstrated that the loss of WRN confers a dramatic increase in the level of phosphorylated RB (Figure. 2) and thus progression into S phase.

Under normal conditions, most cell types cycle through replication maintaining a consistent rate through each phase of the cycle. Of great importance in regulating this rate is the RB protein, which serves as the gatekeeper of the restriction point (R) during the late G1 phase. If this discrete check point is interrupted, the cells lose their most important control mechanism for inhibiting unwanted proliferation [48]. As seen in our study, the increased levels of γRB led to the release of E2F1, a transcription factor that both induces the synthesis of S phase associated proteins and drives the G1/S phase transition, and unregulated accelerated proliferation of HL60 sh-WRN cells. We further showed that increased protein levels of cyclin D3 might be contributing to the elevated γRB level, but we are unsure if this is the sole protein responsible for the increase. Moreover, our data suggests that the presence of normal functioning p53 in TK6 cells may have compensated for the deleterious impacts resulting from the loss of WRN. This implies that WRN's effects on DNA repair and cell cycle stability may be highly modulated by the interaction with other key proteins, in particular p53 [22], [49], [50].

WRN plays a role in RAD51-dependent HR DNA repair, in which WRN is believed to promote intermediate resolution and to suppress cross-over events [27], [51]. This has been thought to be critical for the role of WRN in the maintenance of genomic stability [52]. It has been demonstrated previously that WRN physically and functionally interacts with RAD51 [53] and BLM [54], however, this is the first time that the loss of WRN has been shown to induce significant up-regulation of RAD51 and BLM protein levels in an *in vitro* model. While it remains unclear how this occurs, it is likely that this dramatic increase is related to the accelerated cell proliferation rate present in WRN deficient HL60 cells as RAD51 is maximally transcribed during S phase [55], [56]. This indicates that a complex relationship exists between WRN deficiency and HR DNA repair mechanisms so as to compensate for this loss.

As one of the major DNA repair mechanisms, HR provides high-fidelity template-dependent DSB repair. If left unrepaired, DSBs could cause chromosomal loss or cell death. However, if aberrantly repaired, the result could be much worse, giving rise to mutations and chromosomal rearrangements that could potentially contribute to cancer development [57]. In WS patients, increased levels of chromosomal aberrations have been reported [36], [37], [38], [58], [59], suggesting that the loss of balanced HR regulation may be associated with the development of this disease. One interesting observation from our study is that the increase in DNA damage disrepair mediated by elevated HR activity, due to the loss of WRN, is only observed in HL60 cells but not in TK6 cells. Previous reports noted that DNA DSBs stimulated HR in *p53* mutant cells but not in *p53* wild-type cells [60], [61], suggesting that p53 appeared to repress HR [62]. However, further studies are needed to determine whether the different status of p53 is the causal reason for the observed differences in HR activity and DNA damage disrepair between HL60 and TK6 cells. We further showed that the hyperactivity of HR resulted in a completely altered karyotype of HL60 sh-WRN cells compared to HL60 sh-NSC cells such that the characteristic abnormal double minute chromosomes and aneuploidy typical of HL60 cells were absent in WRN deficient HL60 cells (Figure. 4). However, the hyper-activated HR and higher repair rates resulting from the loss of WRN in HL60 cells led to a higher frequency of SCE, indicating an increased frequency of disrepair and thus genomic rearrangements and gene mutations. This is of particular importance for tumorigensis as the loss or inactivation of WRN may accelerate the mutation of other critical genes, which would thus enhance the likelihood of cancer. More importantly, this gain of function of enhanced disrepair rate confers a growth advantage in WRN deficient HL60 cells lacking sufficient DNA damage response mechanisms mediated by p53 [63], [64]. In addition, previous studies have shown that WRN involved in Non-homologous end joining (NHEJ) mediated DSBs repair pathway by interacting with Ku70/80 [65], [66] and XRCC proteins [67]. WRN appears to play a structural role in optimizing DSBs break repair through the HR and NHEJ repair pathway [28]. While it is beyond the scope of this study, further investigation of the status of NHEJ repair mechanisms in these models is warranted.

**Figure 5:**
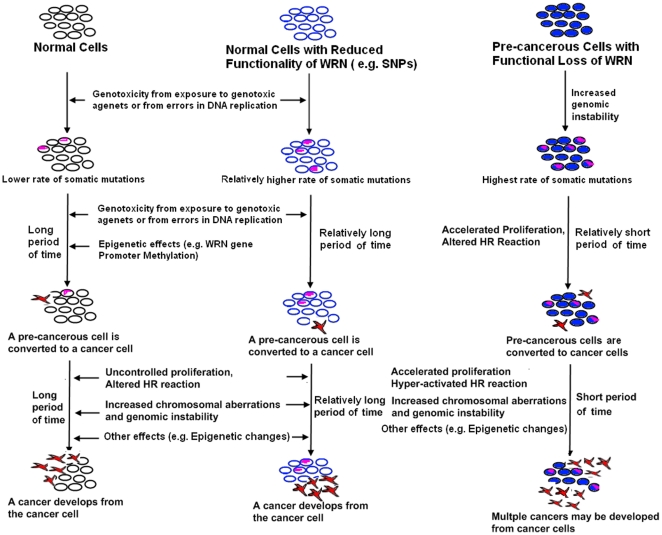
A hypothetical etiological model for cancer development related with WRN deficiency. On the basis of this study and outside literature, a multi-step model for tumor development is proposed in WS patients and other human cell types with reduced functionalities of WRN. We speculate that either complete or partial loss of function of WRN can lead to both increased genomic instability and an increase of somatic mutation rate, which in turn can result in an increased susceptibility for the gain or loss of functions of other genes. The functional loss of WRN in combination with a deficiency in other key regulators of the cell cycle or DNA damage repair pathway may result in an accelerated conversion from pre-cancerous to cancerous cell line, thus allowing for rapid progression of tumorigenesis and cancer formation in WS patients and other diseases related to WRN deficiency.

A multistep model of carcinogenesis is well accepted, in which an initial mutation in a key DNA repair or metabolism gene leads to the accumulation of somatic mutations at a higher frequency. If these somatic mutations occur in other important tumor suppressors or pro-oncogenes, the tumorigenic process is initiated and tumor cells gain the ability to start growing uncontrollably. Our study of silencing *WRN* in HL60 and TK6 cells suggests that this multistep model may accurately illustrate the etiology of cancer development in WS patients as well as other diseases related to functional loss of WRN (Figure. 5). WRN has been proposed as a “caretaker” of the genome [14], and an increased level of chromosomal aberrations has also been reported in WS patients [58], [59]. Thus, functional loss of WRN would in turn result in the loss or gain of function of other genes due to the increased genomic instability. We thus hypothesized that the loss of WRN together with some of its interacting partners would lead to rapid proliferation through the interruption of normal cell cycle regulation, resulting in the upregulation of HR activity and the induction of more chromosomal aberrations and genomic instability that may be responsible for the transformation from precancerous cell to cancerous cell in WS patients. More importantly, this hypothesis can be applied to WRN deficiency in general, in which the combination of WRN deficiency and the functional loss of other critical genes could accelerate this transformation and subsequently promote tumor formation. This is supported by recent studies that showed association between *WRN* polymorphism and risks of cancer development, including, but not limited to breast, gastric adenocarcinoma and bone and soft tissue sarcomas [9], [10], [11], [12]. Moreover, due to the role of WRN in protecting cells against DNA damage, functional loss of WRN in cells with chemically induced DNA damage, such as with benzene, may result in uncontrolled replication, which can result in genomic instability and disease [68], [69].

Although this model provides a reasonable description of WRN's mechanistic role during the development of cancer, we are aware of the potential limitations of this study and the need for further studies. While HL60 and TK6 cells are both hematopoietic cell lines, p53 status is probably only one of the differences between them. Thus, while our data suggests a role of p53 status in the different responses of HL60 and TK6 cells to the silencing of *WRN*, we cannot exclude the contribution of additional factors, given the wide range of interactions that occur between WRN and other cellular proteins [8], [9], [10]. To further investigate the role of p53 in modulating the responses of HL60 and TK6 cells to WRN deficiency, we are currently conducting experiments to re-introduce p53 to HL60 sh-WRN cells and to silence p53 in TK6 sh-WRN cells. With these studies, we aim to determine if the change in p53 status will leads to a reversal of the effects observed in this study, e.g. accelerated growth rate and increased number of chromosomal aberrations induced by highly activated HR in TK6 cells with p53 and WRN double knockdown, and vice versa, in HL60 cells with a functional p53 protein. However, it is clear that additional studies using other p53 wild-type/null cell line variants or cells deficient in other key players, such as RB or its regulators, are necessary to strengthen our hypothesis.

## Materials and Methods

### Cell Culture and chemical treatments

Human hematopoietic HL60 and TK6 cells were obtained from the American Type Culture Collection (Manassas, VA). HL60 cells were grown in IMDM (GIBCO, San Diego, CA) with L-glutamine and 20% fetal bovine serum (Omega Scientific, San Diego, CA). TK6 cells were maintained in RPMI 1640 medium with 10% fetal bovine serum in standard conditions. Hydroquinone (Sigma Aldrich, St. Louis, MO) was dissolved in 1XPBS for all experiments. Cells were treated with 1XPBS and 10, 20, or 50 uM hydroquinone for HL60 cells or 5, 10 and 20 uM hydroquinone for TK6 cells at a cell density of 4–5×10^5^ cells/ml.

### Generation of shRNA and retroviral transduction

The detailed design and sequences of *WRN* shRNA construct (sh-WRN) and non-target shRNA control (sh-NSC) have been previously reported [19]. Briefly, lentiviruses were produced by transfection of 293FT cells with the packaging plasmids along with the lentiviral shRNA vector, according to the manufacturer's instructions (Invitrogen, Carlsbad, CA). HL60 and TK6 cells were transduced and put in selection media containing 6 µg/ml and 8 µg/ml of blasticidin respectively 48 hours post-transduction. Cells resistant to blasticidin were isolated and assayed for protein expression levels using Western blot analysis.

### Cell Proliferation

Control (HL60 sh-NSC and TK6 sh-NSC) and *WRN* knock down (HL60 sh-WRN and TK6 sh-WRN) cell lines were seeded at 2×10^5^ and allowed to grow under normal conditions. Cells were enumerated daily for seven days using the trypan blue assay. Cell proliferation and viability were measured in triplicate in three independent experiments.

### Single-cell gel electrophoresis (COMET)

The alkaline Comet assay was performed as previously described [70] with some modifications. To observe endogenous DNA damage, cells were seeded in culture for 3 days and maintained at a cell density of 5×10^5^ cells/ml. Cells were collected at 6 and 24 h for analysis. To measure chemically induced DNA damage, cells were seeded as above and exposed to hydroquinone for 6 h prior to DNA damage analysis. Five hundred randomly chosen cells per slide were scanned and analyzed automatically using CometScan imaging software (Metasystems, Germany). Cells were subsequently screened manually to exclude cells that did not meet stringent requirements (i.e. poor staining, loss of focus, or oddly shaped). Mean tail length, moment and percentage of tail DNA [71], all measurements of total DNA damage, were calculated for ∼400 cells. All slides were coded to prevent observer bias. Non-parametric method Mann-Whitney U test was used to compare DNA damage between WRN deficient and control cells (*p value <0.05, **p value <0.01).

### Cell Cycle Analysis

For analysis of cell cycle, HL60 and TK6 control and *WRN* knock down cells were cultured in the same manner as for COMET analysis. Cells were collected at 6 and 24 h, washed twice with ice-cold PBS buffer (pH 7.4), fixed with 70% ice-cold ethanol at −20°C for 15 min, stained with propidium iodide (PI) (100 µg/ml) and treated with RNase A overnight. In three independent experiments done in triplicate, at least 1×10^4^ cells were analyzed on a Beckman Coulter EPICS XL-MCL flow cytometer using System II software. The collected data was then analyzed by Flowjo software (Ashland, OR 97520).

### Immunoblot analysis

Total cell lysates were collected from 5×10^6^ cells using 300 uL of radioimmunoprecipitation assay (RIPA) lysis buffer. Nuclear extractions were collected using 1×10^7^ cells and a nuclear extraction kit (Millipore, Billerica, MA) according to the manufacturer's protocol. Protein concentrations were determined by the DC assay (Bio-Rad, Hercules, CA). Equal protein amounts were resolved by sodium dodecyl sulfate polyacrylamide gel electrophoresis (SDS-PAGE), transferred onto nitrocellulose membranes, and probed for WRN (Santa Cruz Biotechnology, Santa Cruz, CA), BLM, RAD51 (Millipore, Billerica, MA), Phospho-Histone H2A.X (γH2AX), P53 Antibody, Phospho-P53 (γP53), p21 Waf1/Cip1, p27 Kip, CDK2, CDK6, Cyclin E, Cyclin D3, Phospho-Rb (γRb), Phospho-CDC2 (γCDC2) (Cell Signaling Technology, Danvers, MA), and β-actin (Sigma-Aldrich, St. Louis, MO). Proteins were visualized using the enhanced chemiluminescence (ECL) method per manufacturer's protocol (Amersham Biosciences, United Kingdom). Film was exposed and developed using the Konica SRX-101 developer (Konica Minolta Medical Imaging USA, Wayne, NJ). Each measured protein except the nuclear protein RAD51 was normalized to β-actin, the loading control, and quantified using ImageJ software (NIH, Bethesda, MD). The Coomassie stain was used according to manufacturer's protocol to confirm loading uniformity of nuclear proteins into the SDS-PAGE gel (Bio-Rad, Hercules, CA), and the gel was subsequently visualized and photographed (Alpha Innotech, San leandro, CA). Data obtained is representative of the averages of at least three independent experiments. Error bars represent SEM.^ *^, *p*<0.05; ^**^, *p*<0.01.

### Non-banding chromosome aberration analysis

Colcemid (0.1 µg/ml, Invitrogen, Carlsbad, CA) was added to each culture 2 hours before harvesting to arrest cells at metaphase. After hypotonic treatment (0.075 M KCl) for 30 min at 37°C, the cells were fixed three times with freshly prepared Carnoy's fixative (methanol: glacial acetic acid  =  3∶1). The fixed cells were dropped onto glass slides, allowed to air dry and stored at −20°C. The cells on the slides were then stained with Giemsa and metaphase spreads were scanned and localized automatically using Metafer software (MetaSystems, Altlussheim, Germany). Metaphases were scored at 1000× magnification to detect numerical and structural chromosomal aberrations. Metaphase spreads were considered scorable if the cells appeared intact with the chromosomes condensed and well spread out as well as if the centromeres and chromatids were readily visible. The structural chromosomal aberrations were defined according to An International System for Human Cytogenetic Nomenclature (2005). All slides for all assays were coded to prevent observer bias.

### Sister-chromatid Exchange Analysis

For SCE analysis, filter sterilized solution of BrdU ((Sigma Aldrich, St. Louis, MO) was added to fresh culture medium at a final concentration of 5 µg/ml and the cells were incubated strictly in the dark for 42 h. Chromosome preparations were then done according to the procedure described for chromosomal aberration frequency measurement. After metaphase were prepared, the slides were incubated 10–15 min strictly in the dark in bisbenzimide H 33258 (Sigma Aldrich, St. Louis, MO) solution (50 µg/ml), rinsed in phosphate buffered water and incubated in fresh phosphate buffered water under UV light source for 60 min. Preparations were rinsed again, incubated for 15 min in preheated 2× SSC at 60°C in the water-bath. After rinsing, preparations were stained in 4% phosphate buffered Giemsa solution. Fifty metaphases were evaluated per treatment level. Only fully differentiated metaphases were evaluated. SCEs were expressed as SCEs/cell. Non-parametric method Mann-Whitney U test was used to compare SCE frequencies between WRN deficient and control cells (*p value <0.05).

### Data Analysis

Statistical analyses of data were performed using one-way analysis of variance (ANOVA). A trend χ2 test was used to compare cell growth trends between control cells and *WRN* knocking down cells. Data obtained is representative of the averages of at least three independent experiments. Error bars represent standard error of the mean (SEM).^ *^, p<0.05; ^**^, *p*<0.01.

## Supporting Information

Figure S1 Rate of double minute positive cells between HL60 sh-NSC and HL60 sh-WRN cells after HQ exposure. The complete disappearance of the characteristic double minute chromosomes was seen in WRN deficient HL60 cells regardless of HQ treatment. HQ treatment alone led to a dose-dependent reduction of double minute chromosomes in HL60 sh-NSC cells.
(0.01 MB TIF)

Table S1 Rate of cells with specific chromosome aberrations (%).
(0.04 MB DOC)
