# Supplementary figures and images for: Comparison of Proliferation and Genomic Instability Responses to WRN Silencing in Hematopoietic HL60 and TK6 Cells
Source: PLoS One. 2011 Jan 18;6(1):e14546. doi: 10.1371/journal.pone.0014546 (PMC3022623; doi:10.1371/journal.pone.0014546)

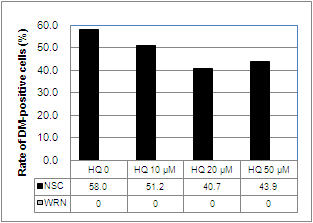

Supplement: Figure S1 — Rate of double minute positive cells between HL60 sh-NSC and HL60 sh-WRN cells after HQ exposure. The complete disappearance of the characteristic double minute chromosomes was seen in WRN deficient HL60 cells regardless of HQ treatment. HQ treatment alone led to a dose-dependent reduction of double minute chromosomes in HL60 sh-NSC cells. (0.01 MB TIF) [file pone.0014546.s001.tif]
