# Supplementary material for: Comparison of Proliferation and Genomic Instability Responses to WRN Silencing in Hematopoietic HL60 and TK6 Cells
Source: PLoS One. 2011 Jan 18;6(1):e14546. doi: 10.1371/journal.pone.0014546 (PMC3022623; doi:10.1371/journal.pone.0014546)
Supplement: Table S1 — Rate of cells with specific chromosome aberrations (%). (0.04 MB DOC) [file pone.0014546.s002.doc]

**Supplement Table 1, Rate of cells with specific chromosome aberrations (%)**

|  | **[HQ] µM** | **cells** | **Chromosome number** | | | | | | **Structural chromosome aberrations** | |
| --- | --- | --- | --- | --- | --- | --- | --- | --- | --- | --- |
|  |  |  | **< 44** | **44** | **45** | **46** | **47** | **> 47** | **including gaps** | **excluding gaps** |
| HL60 sh-NSC | 0 | 193 | 8.3 | 38.3 | 49.7 | 3.6 | 0 | 0 | 0.5 | 2.1 |
|  | 10 | 164 | 1.8 | 42.7 | 51.8 | 3.7 | 0 | 0 | 1.2 | 1.2 |
|  | 20 | 167 | 6.0 | 32.9 | 55.7 | 3.0 | 1.8 | 0.6 | 0.6 | 1.2 |
|  | 50 | 198 | 4.5 | 35.4 | 55.1 | 4.5 | 0 | 0.5 | 0 | 0 |
| HL60 sh-WRN | 0 | 153 | 5.9 | 14.4 | 12.4 | 53.6 | 12.4 | 1.3 | 2.0 | 3.3 |
|  | 10 | 199 | 4.0 | 14.1 | 10.6 | 61.8 | 7.0 | 2.5 | 1.5 | 3.0 |
|  | 20 | 126 | 10.3 | 9.5 | 11.1 | 61.1 | 3.2 | 4.8 | 3.2 | 4.0 |
|  | 50 | 193 | 3.1 | 8.3 | 9.3 | 64.8 | 11.4 | 3.1 | 1.6 | 2.1 |
